# In properly selected patients with differentiated thyroid cancer, antithyroglobulin antibodies decline after thyroidectomy and their sole presence should not be an indication for radioiodine ablation

**DOI:** 10.20945/2359-3997000000123

**Published:** 2019-04-15

**Authors:** Luis Felipe Zavala, María Inés Barra, Roberto Olmos, Michael Tuttle, Hernán González, Nicolás Droppelmann, Lorena Mosso, José M. Domínguez

**Affiliations:** 1 Pontificia Universidad Católica de Chile Departments of Endocrinology Faculty of Medicine Pontificia Universidad Católica de Chile Santiago Chile Departments of Endocrinology, Faculty of Medicine, Pontificia Universidad Católica de Chile, Santiago, Chile; 2 Department of Endocrinology Service Memorial Sloan-Kettering Cancer Center New York United States Department of Endocrinology Service, Memorial Sloan-Kettering Cancer Center, New York, New YorkUnited States; 3 Pontificia Universidad Católica de Chile Faculty of Medicine Pontificia Universidad Católica de Chile Santiago Chile Head and Neck Surgery, Faculty of Medicine, Pontificia Universidad Católica de Chile, Santiago, Chile

**Keywords:** Thyroid cancer, antithyroglobulin antibodies, radioiodine ablation

## Abstract

**Objective:**

Our objective was to evaluate the trend of antithyroglobulin antibodies (TgAb) during follow-up of patients with differentiated thyroid cancer (DTC) treated without RAI, as well as their role in the risk of recurrence.

**Subjects and methods:**

This was a prospective, descriptive study. A total of 152 consecutive patients with DTC treated in a single institution undergoing total thyroidectomy without RAI and followed for a median of 2.3 years (0.5-10.3) were divided in two groups: TgAb(-) (n = 111) and TgAb(+) (n = 41). Patients were classified according to AJCC 7^th^ and 8^th^ editions, as well as to their risk of recurrence and response to treatment categories.

**Results:**

Both groups, TgAb(-) and TgAb(+), were similar regarding patient and tumor characteristics. At the end of follow-up, 90 (59.2%), 57 (37.5%), 3 (2%) and 2 (1.3%) patients achieved excellent, indeterminate, biochemically incomplete and structurally incomplete response, respectively. The risk of structural recurrence was similar in both groups (TgAb[-] 0.9% vs. TgAb[+] 2.4%, p = 0.46). In the TgAb(+) group, TgAb became negative in 10 (24.4%), decreased ≥ 50% without negativization in 25 (60.9%), decreased < 50% in 4 (9.8%) and remained stable or increased in 2 (4.9%) cases. The only incomplete structural response had increasing TgAb during follow-up.

**Conclusions:**

In properly selected patients with DTC, TgAb concentration immediately after total thyroidectomy should not mandate RAI ablation, and their trend during follow-up may impact the risk of recurrence.

## INTRODUCTION

Differentiated thyroid cancer (DTC) is the most common endocrine malignancy, and its incidence has increased in the last decades due, in part, to expanded availability of neck ultrasonography (US) (1-3). Because most of these newly diagnosed tumors have low risk of recurrence, there is a need to personalize their management to minimize the adverse effects of therapy (4).

The treatment of DTC includes surgery, selective use of radioactive iodine (RAI) and levothyroxine supplementation (4). RAI has proven to be effective in patients with intermediate and high risk of recurrence, while its utility has been questioned in low-risk patients (5). Because one of the benefits of RAI is to facilitate patient follow-up, its use has been suggested in the presence of elevated antithyroglobulin antibodies (TgAb) to enable correct interpretation of serum thyroglobulin (Tg) (6). However, RAI use is associated with short- and long-term adverse effects, which can significantly impair patients’ quality of life. For this reason, the indication of RAI must be adjusted to the patient’s individual risk of recurrence and mortality (7-9).

After initial treatment, follow-up of patients with DTC includes measurement of serum Tg and imaging studies according to the initial risk of recurrence (10-12). About 20% of patients have TgAb(+), which can interfere with Tg measurement, usually underestimating their concentration and thereby potentially leading to false-negative results (13-17). For this reason, most studies evaluating treatment and follow-up of patients with DTC (with or without RAI) exclude TgAb(+) cases (18). The TgAb are not direct tumor markers, and their concentration reflects the immune response to changes in the Tg antigen, secondary to benign or malignant disease (19). To estimate real changes in their concentration, TgAb usually require a minimum follow-up of 6 months, during which the trend in their concentration appears to be more accurate as a marker of disease rather than the absolute level itself (19). For instance, in patients with DTC and TgAb(+) treated with RAI, a reduction greater than 50% in TgAb concentration during the first 12 months of follow-up has been associated with a better prognosis (20,21). Matrone and cols. recently published a prospective follow-up of TgAb(+) patients with microcarcinomas treated without RAI (22). To the best of our knowledge, no other prospective study has compared the clinical outcome of patients with DTC treated without RAI based on the presence or absence of TgAb.

The aim of this study was to prospectively evaluate the trend of TgAb concentration during follow-up of patients with DTC treated without RAI, as well as their impact on the risk of recurrence and response to treatment.

## SUBJECTS AND METHODS

### Subjects

This study was approved by the Ethics Committee of Pontificia Universidad Católica de Chile.

Patients 18 years old or older, consecutively treated and followed prospectively at our institution between December 2012 and December 2017, who met the following criteria were initially selected: (i) diagnosis of DTC; (ii) subjected to total thyroidectomy, with neck dissection performed only with evidence of clinically apparent nodal disease detected at clinical examination, preoperative neck US or during intra-operative inspection by the surgeon; and (iii) followed for at least 6 months with at least 2 measurements of Tg and TgAb. Nodal disease was classified in low and high volume according to 2015 ATA guidelines (4).

The need for RAI was defined in each patient following the recommendations of the 2015 ATA guidelines (4). Patients were classified according to ATA recurrence risk category (low, intermediate and high) as well as the 7^th^ and 8^th^ editions of the AJCC/UICC staging system (I, II, III and IV) based on the preoperative neck US, intraoperative findings and final surgical pathology report (23).

Patients that met any of the following criteria were excluded: (i) partial thyroidectomy; (ii) absence of or incomplete surgical pathology report; (iii) follow-up of less than 6 moths; and (iv) Tg, TgAb or US not performed during follow-up. From the 180 initially included patients, 28 were excluded due to of lack of follow-up (n = 13) or insufficient clinical and pathological information (n = 15).

### Follow-up

After initial surgery, all patients were treated with a levothyroxine (LT4) dose sufficient to maintain a serum thyrotropin (TSH) concentration < 1.0 UI/mL. The patients underwent clinical examination, Tg, TgAb and neck US every 6 months during the first year and thereafter at 6- to 12-month intervals at their attending physician’s discretion.

### Outcomes

Cervical neck US was performed by experienced radiologists. Negative neck US was defined as either normal exploration or the presence of any of the following findings: (i) thyroid bed avascular nodules ≤ 5 mm in diameter, stable during follow-up or (ii) reactive cervical lymph nodes. Disease recurrence/persistence was defined as suspicious findings in images, confirmed with cytopathologic study and Tg measurement on aspirate.

Response to treatment was defined according to US, Tg and TgAb findings during follow-up. Excellent response was defined when all of the following were present: negative US, Tg < 0.2 ng/mL and negative TgAb. Incomplete biochemical response was defined in the presence of negative US and either non-stimulated Tg > 5 ng/mL plus negative TgAb, or positive TgAb with an increase in concentration. Incomplete structural response was defined as highly suspicious neck US (or any other image modality), confirmed through cytological study, regardless of Tg or TgAb serum concentration. Indeterminate response was defined either as (i) negative neck US plus Tg ≥ 0.2 - ≤ 5 ng/mL plus TgAb(-) or (ii) negative US plus TgAb(+) with decreasing or stable concentration during follow-up or (iii) nonspecific findings through imaging studies (24). To refine the evaluation of the impact of TgAb on prognosis, we classified the TgAb concentration decrease during follow-up as < 50% or ≥ 50%.

### Assays

Tg was measured using a chemiluminescent immunoassay with functional sensitivity of 0.1 ng/mL (Elecsys II, Roche Diagnostics, Rotkreutz, Switzerland). TgAb were also measured using a chemiluminescent immunoassay (Architect i1000, Abbot Laboratories, Abbott Park, IL), with a reference value of up to 4.11 IU/mL and analytical sensitivity of 1.0 IU/mL. For the purpose of this study, every value below 4.1 UI/mL was considered negative. Both Tg and TgAb were normalized with CRM 457.

### Statistical analysis

Continuous variables are presented either as mean and standard deviation or median with range, as appropriate. Categorical comparisons were performed using Fisher’s exact test, and continuous variables were compared using Student’s t-test. A p-value of < 0.05 was considered significant. Statistical analysis was performed using SPSS (v.15.0.0: SPSS, Inc., Chicago, IL).

## RESULTS

A total of 152 patients with a mean age of 40.5 (18-80) years, followed for a median of 2.3 (0.5-10.3) years were included. Total thyroidectomy alone was performed in 124 (81.6%) patients, and total thyroidectomy with lymph node dissection in 28 (18.4%). Papillary thyroid cancer was the histological diagnosis in 150 (98.7%) and follicular thyroid cancer in 2 (1.3%), both cases minimally invasive and less than 4 cm. Of the total cohort, 97 (64%) had microcarcinoma, 54 (35.5%) were multicentric, 40 (26.3%) were bilateral and 13 (8.5%) had lymph node metastases: 8 with low-volume and 5 with high-volume disease, with highest nodal diameter of 0.5 cm and no cases of extra-nodal extension (Table 1).

**Table 1:** Characteristics of the 152 patients studied

	Total cohort (N = 152)	Antithyroglobulin Antibodies		p-value

		Negative (n = 111)	Positive (n = 41)	
Age (years)	40.5 (18-80)	42 (18-80)	35 (18-63)	0.25
Follow-up (years)	2.3 (0.5-10.3)	2.3 (0.5-10.3)	2.3 (0.5-9.8)	0.87
Microcarcinoma	97 (64%)	72 (75%)	25 (61%)	0.70
AJCC VII edition				
I	138 (90.8%)	99 (89.2%)	39 (95.2%)	0.375
II	3 (2%)	2 (1.8%)	1 (2.4%)	
III	11 (7.2%)	10 (9%)	1 (2.4%)	
AJCC VIII edition				1
I	152 (100%)	111 (100%)	41 (100%)	
ATA (2015)				
Low risk	123 (80.9%)	90 (81.1%)	33 (80.5%)	0.93
Intermediate risk	29 (19.1%)	21 (18.9%)	8 (19.5%)	

According to the ATA 2015 guidelines, 123 (80.9%) and 29 (19.1%) patients were classified as low and intermediate risk of recurrence, respectively. No patients were classified as high risk of recurrence. According to the AJCC 7^th^ edition, 138 (90.8%) patients were classified as stage I, and according to the AJCC 8^th^ edition, 100% of the cohort was classified as stage I (Table 1).

A median of 4.4 (2.0-7.0) Tg and TgAb measurements were performed. Forty-one (27%) patients had TgAb(+) at diagnosis. Their clinical characteristics were similar to patients with TgAb(-) including age, proportion of microcarcinomas and cases classified either as stage I according to the AJCC 7^th^-8^th^ editions or low risk of recurrence according to the ATA 2015 guidelines (Table 1).

During the study, 2 (1.3%) patients of the whole cohort developed structural incomplete response, while 102 (67.1%) patients achieved excellent response at some point during follow-up. Of this last subgroup, no patients developed structural disease, while at the end of follow-up, 90 (88%) patients sustained excellent response and 12 patients were classified as having indeterminate response (Table 2). Of the 95 patients with initial indeterminate response, 53 (55.8%) developed excellent response during follow-up at some point, while at the end of the study, 46 (48.4%) achieved excellent response, 46 (48.4%) indeterminate response, 2 (2.1%) biochemical incomplete response and 1 (1.1%) structural incomplete response (Table 2).

**Table 2:** Clinical outcomes in the whole cohort and according to the presence of anti-thyroglobulin antibodies

	Total cohort (n = 152)	Anti-thyroglobulin antibodies		p-value

		Negative (n = 111)	Positive (n = 41)	
Structural incomplete response				
Yes	2 (1.3%)	1 (0.9%)	1 (2.4%)	0.46
No	150 (98.7%)	110 (99.1%)	40 (97.6%)	
Response to treatment at the end of follow-up				
Excellent	90 (59.2%)	80 (72.1%)	10 (24.4%)	
Indeterminate	57 (37.5%)	29 (26.1%)	28 (68.3%)	< 0.01
Biochemical incomplete	3 (2%)	1 (0.9%)	2 (4.9%)	
Structural Incomplete	2 (1.3%)	1 (0.9%)	1 (2.4%)	

The rate of incomplete structural response was similar between TgAb(-) and TgAb(+) patients: 0.9% vs 2.4% (p = 0.46) (Table 2). Regarding the 41 patients with TgAb(+), TgAb concentration became negative in 10 (24.4%), decreased ≥ 50% without negativization in 25 (60.9%), decreased < 50% in 4 (9.8%) and remained stable or increased in 2 (4.9%) cases (Table 3). Among the patients in whom TgAb concentration decreased, the median time to reach its nadir was 1.9 years (range 0.2-3.1; interquartile range 1.5-3.0). At the end of follow-up, 10 (24.4%) patients achieved excellent, 28 (68.3%) indeterminate, 2 (4.9%) biochemical incomplete and 1 (2.4%) structural incomplete response. In this group, the only incomplete structural response had increasing TgAb during follow-up.

**Table 3:** Clinical outcome of patients with positive antithyroglobulin antibodies

Trend of TgAb concentration during follow-up	N (%)	Structural disease
Negativization	10 (24.4%)	0 (0%)
≥ 50% decrease without negativization	25 (60.9%)	0 (0%)
< 50% decrease	4 (9.8%)	0 (0%)
Stable or increase	2 (4.9%)	1 (50%)

TgAb: antithyroglobulin antibodies.

The three patients with incomplete biochemical response underwent active surveillance, and none developed structural disease at the end of follow-up. In the two patients with a structural incomplete response, both had US/cytologically proven cervical nodal disease at 6 and 18 months after initial therapy. These patients underwent neck dissection and RAI and were both free of disease at the end of follow-up.

## DISCUSSION

This study prospectively evaluated the clinical and biochemical evolution of patients with DTC treated with total thyroidectomy without RAI, showing that most of them (59.2%) achieved excellent response to treatment at the end of follow-up, and only 1.3% developed structural incomplete response that required further therapy. Our cohort included patients with DTC similar to most previous studies that included patients treated without RAI: predominance of papillary thyroid cancer of initial low risk of recurrence, as well as a similar rate of biochemical and structural recurrence during follow-up (25,26).

As in other studies, 27% of our patients had TgAb(+) at diagnosis, with a similar clinical presentation and evolution to those with TgAb(-), including a comparable risk of incomplete structural response during follow-up. Our findings are consistent with other reports that evaluated the impact of TgAb on DTC, with their presence not being associated with a higher risk of recurrence or mortality (27), as well as some studies suggesting that their concentration is not related to tumor mass (19,27).

It has been previously reported that in patients with DTC and TgAb(+), the trend of the TgAb concentration during follow-up can be used as a surrogate marker of Tg when evaluating the response to treatment (28). Unfortunately, this information is derived from retrospective studies including patients initially treated with RAI (20). Our study exclusively included patients treated without RAI followed prospectively. In the AcTg(+) group, the trend in antibodies concentration during follow-up was favorable in 85.3% of cases, with negativization in 24.4% and a decrease of ≥ 50% without negativization in another 60.9%. In this subgroup, there were no cases of structural recurrence, and 24.4% of patients achieved excellent response at the end of follow-up (negative neck US plus TgAb[-] and Tg < 0.2 ng/mL). These findings are consistent with studies that included patients treated with RAI, in which a decrease ≥ 50% in TgAb concentration was associated with a risk of recurrence of 0 and 3.3% in low- and intermediate-risk patients, respectively (20,29). Also similar to our cohort, previous studies focusing on patients with TgAb(+) reported a ≥ 50% decrease of their concentration in 75%, with negativization in 30% of cases (28,30). The similar behavior of TgAb in patients treated with or without RAI casts doubt on the role of RAI to facilitate follow-up after initial treatment.

Interestingly, at the end of follow-up, 75.6% of the whole cohort achieved either one of the following: i) excellent response to therapy (including initially TgAb[-] and TgAb[+] with negativization during follow-up) or ii) initially TgAb(+), with ≥ 50% decrease of TgAb without negativization, in addition to Tg < 0.2 ng/mL and negative US. It is well known that both aforementioned conditions are associated with a very low risk of recurrence (20). As mentioned before, 27% of patients with initial TgAb(+) achieved excellent response at the end of follow-up. However, a significant decrease in TgAb concentration, even without negativization, is also associated with an excellent prognosis. Our data, hopefully confirmed by further studies with longer follow-up, may challenge the current definition of excellent response, allowing for the inclusion of patients with TgAb(+), in which antibodies concentration decrease ≥ 50% (in conjunction with a negative US) during follow-up. These findings confirm that, in properly selected patients with low or intermediate risk of recurrence, in whom the use of RAI is selective, the behavior of Tg and TgAb during follow-up can modulate the indication for RAI. Furthermore, previous studies have shown that in low- and intermediate-risk patients, the efficacy of RAI is preserved even if administered up to one year after surgery, giving the clinician enough time to thoroughly decide its indication (31-33).

It is also notable that of the 95 patients with an indeterminate response at their first follow-up evaluation (representing 63% of the entire cohort), only 1 of them developed structural disease, which was successfully treated with surgery and RAI. This finding, consistent with previous reports, suggests that in patients with DTC of low and selected intermediate risk, a conservative approach with active surveillance is safe and avoids exposure of an important number of patients to therapies with potential risks, such as RAI and surgery (25).

As previously described, most patients in our study achieved either excellent or indeterminate response. However, and despite the limited number of cases, half of the patients with stable or increasing TgAb(+) concentration during follow-up developed structural recurrence. Our finding, similar to previous reports, highlights the importance of closer surveillance in this specific group of patients (25).

One limitation of this study was the limited length of follow-up (median 2.3 years), which could result in underestimation of the risk of disease recurrence. However, 75.6% of our cohort achieved either excellent response or negative US plus a significant decrease in TgAb levels, findings that in a previous study from Momesso were associated with a risk of recurrence of only 1.3% after a median follow-up of 8.3 years (25). A second limitation was the high proportion (64%) of microcarcinomas in our cohort, considering that according to the 2015 ATA guidelines, these tumors could be submitted to active surveillance with neck US, in the absence of sonographic or cytologic signs of aggressiveness. However, in our study, we began recruitment in 2013, years before the publication of the aforementioned guidelines, when the indication of fine-needle aspiration was mainly driven by clinical and sonographic risk factors, regardless of nodule size (34).

Finally, there is still doubt concerning the definition of TgAb(+), with values above both the analytical sensitivity as well as the upper reference limit of the assay being considered TgAb(+). Regarding this point, a recent study did not find differences in clinical outcomes when comparing patients with TgAb < 1.0 IU/mL and TgAb < 4.11 IU/mL using the same assay as in our present study (35).

We conclude that in properly selected patients with DTC, the concentration of TgAb usually declines during follow-up after total thyroidectomy, and their sole presence should not be an indication of RAI ablation.
